# Determination of nasal carriage and skin colonization, antimicrobial susceptibility and genetic relatedness of *Staphylococcus aureus* isolated from patients with atopic dermatitis in Szczecin, Poland

**DOI:** 10.1186/s12879-021-06382-3

**Published:** 2021-07-23

**Authors:** Helena Masiuk, Aleksandra Wcisłek, Joanna Jursa-Kulesza

**Affiliations:** Independent Laboratory of Medical Microbiology, Pomeranian Medical University, al. Powstańców Wielkopolskich 72, 70-111 Szczecin, Poland

**Keywords:** *Staphylococcus aureus*, Atopic dermatitis, Skin and soft tissue infections, Nasal carriage

## Abstract

**Background:**

Atopic dermatitis (AD) is one of the most frequent chronic and inflammatory skin condition. AD is characterized by damaged epidermal barrier, xerosis and pruritus of eczematous skin lesions which tend to flare. The duration and frequency of exacerbation of AD symptoms markedly affects the quality of patient life. AD results from the interplay between host genetics, immunity, and environmental factors, however the detailed pathogenesis of this disease is still not entirely cleared. Furthermore, disturbances of the skin microbiota and skin functional impairment predispose to secondary skin infections. *Staphylococcus aureus* colonizes skin and mucous membranes of 20 to 80% of healthy individuals and of 90% of patients with AD in whom this bacterium is accounted as an important AD exacerbating factor. It is also proven, that *S. aureus* nasal carriage significantly increases the risk for self-transmission and endogenous infection. In the current study the presence of *S. aureus* either in nasal vestibule and on lesioned skin of 64 patients with AD enrolled in 10-year autovaccination program was determined. The genetic relatedness of 86 *S. aureus* isolated from patients nose and skin using Pulsed Field Gel Electrophoresis (PFGE) and antimicrobial susceptibility of all strains to methicillin, erythromycin, clindamycin, mupirocin, gentamicin, amikacin, tetracycline, chloramphenicol and cotrimoxazole was also evaluated.

**Results:**

In total 23 PFGE genotypes and 24 unique patterns were distinguished. 34 patients were *S. aureus* nasal carriers. Simultaneous presence of *S. aureus* in nose and on affected skin was found in 16 carriers colonized by indistinguishable or potentially related *S. aureus* vs 2 carriers colonized with non-related *S. aureus* in nasal vestibule and on skin.

4 isolates were methicillin resistant (MRSA) among which 3 showed constitutive MLSB resistance phenotype and remaining one was resistant to tetracycline and chloramphenicol.

In 4 isolates inducible MLSB resistance phenotype was found, one of them was additionally resistant to tetracycline. 7 *S. aureus* were mupirocin resistant among them 3 - isolated from one patient, were resistant simultaneously to tetracyclines and chloramphenicol. 7 strains demonstrated resistance to chloramphenicol and susceptibility to all tested antimicrobial agents. The susceptibility to gentamicin, amikacin and cotrimoxazole among all examined *S. aureus* was confirmed.

**Conclusion:**

The obtained results indicated non-clonal structure of *S. aureus* circulating in AD patients. PFGE results showed the clonal-structure of vast majority of *S. aureus* isolated from nose and skin from nasal carriers what may prove the autoinfection in these patients. All examined patients the moderate or strong severity of AD was reported. Susceptibility to most antibiotics among isolated strains was also observed.

## Background

Atopic dermatitis – AD (also called atopic eczema AE) is a long - standing inflammatory dermatosis characterized by intense itching of affected skin and recurrence of chronic eczematous lesions [1]. In adult patients AD mainly manifests with lesions localized mostly on face and neck, and in up to 30% of these patients the hand atopic eczema is determined [2].

The multifactorial pathogenesis of AD includes altered immune response, several genetic predispositions and environmental factors which along with epidermal barrier dysfunctions significantly impair the functional integrity of the skin [3]. During flares of the disease the disturbances of skin microbial composition and also other skin barrier defects consequently contribute to exacerbation of AD symptoms [1].

*Staphylococcus aureus* (*S. aureus*) is the major pathogenic bacterium which activity is closely associated with severity and the course of the AD [4]. Although, *S. aureus* is present on skin and mucous membranes of healthy individuals and remains in balance with the host, its overgrowth is most probably linked to the reduced number of skin microbiota representatives on affected skin normally inhibiting activity of this bacterium. Nevertheless, some authors have suggested, that composition of the whole skin microbiome correlates with the course of AD and exacerbation of its symptoms [5–7]. *S. aureus* isolated from individuals with AD demonstrates enhanced ability to adhere to corneocytes and produces toxins and enzymes contributing to the exacerbation of disease symptoms [8].

Higher rates of *S. aureus* nasal colonization, strongly linked with the duration and severity of AD have been observed in patients with AD. Moreover, patients persistently colonized by *S. aureus* in nose are more prone to skin and soft tissue infections (SSTIs), and therefore experience more often the AD symptoms worsening [9–12].

In the current study the relatedness of 86 *S. aureus* isolated from nose and skin of patients with atopic dermatitis was determined with the special regard to genetic relatedness of strains from nose and skin in *S. aureus* nasal carriers. Obtained results indicate the autoinfection with *S. aureus* in nasal-carriers. The results of antimicrobial susceptibility test proved the good susceptibility in vast majority of strains to antimicrobial drugs recommended for treating AD complicated by *S. aureus* presence.

## Methods

The aim of the study was to determine the genetic relatedness of 86 *S. aureus* strains isolated from affected skin and nose of 64 non-related patients with AD and to determine the susceptibility of isolated strains to antimicrobial agents.

### Study population

A total of 64 non-related patients with chronic AD were enrolled in the study. Eligibility for the autovaccination program is voluntary but patients must meet certain criteria including the diagnosis of AD made by a dermatologist. A referral for an autovaccine from dermatologist is an absolute requirement. The diagnosis is based i.a. on physical examination and includes The assessment of AD severity is scored by dermatologist in accordance with SCORAD (Scoring Atopic Dermatitis) index. The duration, severity of the AD and frequency of the disease exacerbations is also evaluated. The first indication for a patient to undergo autovaccine therapy is the chronicity of the disease and documented unsatisfactory effectiveness of all previous forms of treatment. In all patients included in the study the AD was considered as the lifelong chronic condition. In all patients the previous treatment with antihistamines, corticosteroids and topical anti-inflammatory preparations was reported.

An autovaccine, called also autologous vaccine, is derived from an inactivated bacterial strain considered as an etiological agent of the infection and intended for use just by the patient from whom the strain was isolated. The intention of autovaccination used in therapy of AD is to stimulate or modulate innate and specific immune response against the *S. aureus* and therefore to relieve the AD symptoms [13]**.** The preparation of autologous vaccines is described in previously published work [14].

Prior the collection of specimens the data concerning the existence of any hypersensitivity/conditions from all patients were collected. All patients were also asked to rate the severity of AD exacerbation at the moment of collection and to evaluate the frequency of exacerbation episodes in previous 6 months. No other data were collected.

The nasal carriage screening allowed to divide examined symptomatic AD patients into 3 groups: nasal carriers with skin actually affected with *S. aureus* (*group I* – 18 patients – 40 strains), nasal carriers with skin actually not affected with *S. aureus* (*group II* – 16 patients - 16 strains) and nasal non - carriers with skin actually affected with *S. aureus (group III – 30 patients -* 30 strains*).*

The median age of patients was 43 years. Females accounted for 57,8% (37/64) vs for 42,2% (27/64) of males**.** All patients manifested the exacerbations of AD symptoms when conducting the examination.

### Bacterial strains

Specimen collection and identification of the microorganism was performed in accordance to the routine microbiological diagnostics procedures. To determine the presence of *S. aureus,* a swab from affected skin and from nose of every patient were obtained. Specimen was collected from each patient from the actually most inflamed skin site*.* All swabs were cultured onto Columbia Agar with 5% of sheep blood (bioMerieux, France) and Mannitol - Salt Agar (bioMerieux, France) and incubated for 18 h in 37 °C. Determination of *S. aureus* species was performed on the basis of ability to ferment mannitol, to coagulate rabbit plasma and on biochemical properties (VITEK System, bioMerieux). The isolated strains were also subsequently used in the preparation of the autovaccines.

### DNA isolation

Analysis of genetic relatedness of all investigated strains was performed using restriction of total bacterial DNA with *SmaI* enzyme (MBI Fermentas, Canada) and separation of restricted fragments using Pulsed Field Gel Electrophoresis (PFGE) according to protocol of Centers for Disease Control and Prevention [15] with some modifications.

After 24 h of incubation on Columbia Agar with 5% of sheep blood (bioMerieux, France) a single colony of each analyzed strain was transferred to 10 ml of Tryptic Soy Broth (TSB) liquid medium and incubated overnight with shaking at 37 °C. After incubation, 90 μl of each culture was centrifuged in 2 ml Eppendorf Tube (Eppi) at 12.000 x g for 2 min in room temperature (RT). After discarding the supernatant bacterial pellet was resuspended in 300 μl TE buffer (10 mM TRIS, 1 mM EDTA, pH 8,0) and incubated in water bath at 37 °C for 10 min. Next, 4 μl of lysostaphin (stock solution 1 mg/ml, 20 mM sodium acetate, pH 4,5; Sigma Aldrich, Germany) and 10 μl of lysozyme (DNA, Gdańsk, Poland) were added. Simultaneously, 1 ml of 2% agarose previously preheated to 55 °C was added to each sample, pipetted, and immediately transferred using spatula into plug molds. Samples were left to solidify at RT. Next, agarose plugs were transferred into 2 ml Eppi containing 1 ml EC buffer (6 mM TRIS HCl, 1 M NaCl, 100 mM EDTA, 0,5% Brij-58, 0,2% sodium deoxycholate, 0,5% *sodium lauroyl* sarcosinate), 25 μl of Proteinase K (DNA, Gdańsk, Poland) and 4 μl of lysostaphin (stock solution 1 mg/ml, 20 mM sodium acetate, pH 4,5; Sigma Aldrich, Germany). The mixture was incubated in water bath at 37 °C for 24 h, washed 4 times with TE buffer (10 mM TRIS, 1 mM EDTA, pH 8,0) in RT and stored in TE buffer at 4 °C till restriction digestion.

### Restriction digestion

Each plug was removed from Eppi using a spatula and placed on a sterile Petri dish. A slice of plug to desire comb size was cut using scalpel, placed in 1,5 ml Eppi containing 500 μl buffer - water mixture (MBI Fermentas, Canada) (10X Buffer Tango stock diluted 1:10 with sterile type I water) and incubated at RT for 30 min. After equilibration the buffer - water mixture was removed by aspirating the buffer with pipet and 300 μl of buffer - water mixture with 3 μl of *SmaI* enzyme were added. The samples were mixed by gently tapping the tubes and incubated in water bath at 30 °C for 24 h. After incubation all samples were washed 3 times with TE buffer. Meanwhile, the TBE (Tris/Borate/EDTA) buffer (Inno - Train Diagnostik GmbH, Germany) and 1% agarose gel were prepared. Enzymatically digested plug slices were loaded into agarose gel wells and two standards were loaded into first and the last well (BioLine, England). The samples were electrophoresed using CHEF - DR apparatus (Bio - Rad Laboratories, France). The parameters were set as follows: run time - 20 h, initial switch time of 5 s and final switch time of 40 s, temperature at 14 °C, voltage at 6 V/cm and the included angle at 120 °C. After running, the gel was stained with ethidium bromide water solution (1:10 with distilled water) in a covered container for 20–30 min. The gel was distained in fresh distilled water 2 times.

### PFGE fingerprints analysis

The gel was visualized under UV light and documented using Quantity One (Bio - Rad Laboratories, France). Digital images were analyzed with FPQuest Software 4.5 (Bio - Rad Laboratories, France). The dendrogram was generated using the Dice correlation coefficient and the unweighted pair group method with arithmetic mean with 1% tolerance and 1% of band position. PFGE band patterns with ≥72% were considered and clonally related (cut off point - Sab = 72%).

### Antimicrobial susceptibility test

The disc diffusion method was performed on Mueller-Hinton agar plates (bioMerieux) with following antibiotic paper discs - disc content (μg): cefoxitin (30 μg), erythromycin (15 μg), clindamycin (2 μg), mupirocin (10 μg), gentamicin (10 μg), amikacin (10 μg), tetracycline (30 μg), chloramphenicol (30 μg), trimethoprim/sulfamethoxazole (cotrimoxazole) (1.25/23.75 μg), (Becton Dickinson, USA). Tests were performed and interpreted according to the guidelines of the European Committee of Antimicrobial Susceptibility Testing (EUCAST) [16].

## Results

### PFGE results

The genetic relatedness of strains isolated simultaneously from affected skin and from nose of *S. aureus* carriers *(group I)*, as well as relatedness of all *S. aureus* isolates were determined. Examined *S. aureus* represented 23 PFGE genotypes (clusters denoted with the letters from A - T and V - X) and 24 unique PFGE genotypes (denoted with the letter U).

**Fig. 1:**
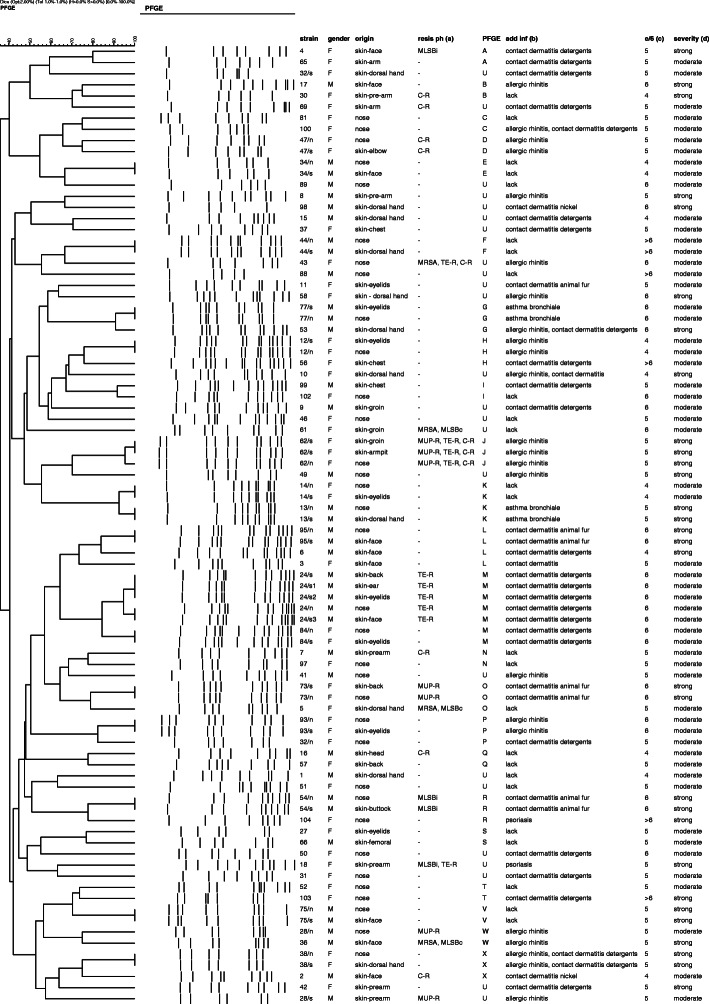
Dendrogram representing the relatedness of 86 *S. aureus* isolated from affected skin and from nose of 64 patients with AD. (Cut-off point – 72.0 (Dice), resistance phenotypes of all examined *S. aureus* and additional patient’s data. The clusters were marked with the letters A-T and V-X, and the unique genotypes were marked with the letter U. Patient/s skin isolate, patient/n – nose isolate. F-female, M-male. (a) – resistance phenotypes - MRSA-methicillin resistant *Staphylococcus aureus*, MLSB-macrolide, lincosamide and streptogramin B resistant (MLSBc-constitutive, MLSBi-inducible), MUP-R – mupirocin resistant, TE-R- tetracycline resistant, C-R – chloramphenicol resistant. (b) additional data concerning the concomitant conditions in examined patients include psoriasis, bronchial asthma, allergic rhinitis, contact dermatitis. Lack – patients who denied the existence of hypersensitivity/conditions. (c)  number of flares in previous 6 months. (d) – exacerbations classified as moderate or strong based on the patients personal rating

Patients from *group I* and *group II* were nasal carriers of *S. aureus* (34 patients in total). In 16 *S. aureus* carriers (*group I*) colonization of affected skin with indistinguishable or potentially related strains was found. In two patients from *group I S. aureus* isolated from nose and skin showed no genetic relatedness and represented different PFGE types. Strains isolated from skin and nose of patients from *group I* within each pair shared the same antibiotic susceptibility patterns. The genetic relatedness analysis was performed in accordance to Tenover’s guidelines [17]. Dendrogram representing PFGE genotypes is shown in Fig. 1.

### Antimicrobial susceptibility test

In total 86 strains of *S. aureus* from 64 patients with atopic dermatitis were tested for antimicrobial drug resistance. 59 isolates (59/86) were susceptible to all tested antimicrobial agents. Methicillin resistance (MRSA) was confirmed in 4 strains. Among MRSA 3 strains showed the presence of constitutive MLSB (MLSBc) and one was simultaneously resistant to tetracycline and chloramphenicol. In 4 strains inducible MLSB (MLSBi) was confirmed and one of them was resistant to tetracycline. Mupirocin resistance was confirmed in 7 strains – among them 3 demonstrated resistance to tetracycline and chloramphenicol (all 3 were isolated from one patient). Resistance to chloramphenicol and susceptibility to other antimicrobial agents was found in 7 strains, resistance only to tetracycline in 5 strains (isolated from one patient). The susceptibility to gentamicin, amikacin and cotrimoxazole among all examined *S. aureus* was confirmed. Distribution of resistance phenotypes among *S. aureus* is shown in Fig. 1.

## Disscussion

The course of atopic dermatitis (AD), an inflammatory skin condition, associated with type I allergic diseases is strongly influenced by a complex of variety of factors such as individual genetic and immunological predispositions, impaired skin barrier function and environment. All these taken together exacerbate AD symptoms from moderate to severe [18], nevertheless the detailed pathophysiology of AD still remains unclear. It is worth to note, that AD has an age-dependent distribution and affects around 20% of children worldwide. In 90% of patients AD begins from an early age, what is most probably associated with higher rates of skin colonization by *S. aureus* in children [19] and in 10% of AD patients it may persist for life [20].

Human skin microbiota is represented by a wide range of microorganisms which coexistence and integrity prevents skin from bacterial invasion. Most of the microorganisms reside on the skin in asymptomatic manner, among them also *S. aureus* which constitutes an integral part of skin microbiome in around 30% human population representatives [21]. It is worth emphasizing that less diverse skin microbiome, observed in patients with AD, may predispose to *S. aureus* overgrowth [22]. Lesional skin colonization by *S. aureus* is commonly observed in AD patients however, colonization may also indicate non-lesional skin [23–25]. It has been demonstrated that the progression and severity of AD are closely related with the ability of *S. aureus* to penetrate through skin barrier what stimulates the immune response leading to the persistent skin inflammation [22]. Dehydrated and thinner skin of AD patients is much more vulnerable to injury and therefore to infection and irritation by allergens [26]. Celakovska and Bukac [27, 28] observed the significant relation between the severity of AD and sensitization mites, animal dander, dust of feather. According to authors observation patients with strong or moderate form of AD suffer more often from sensitization to aboved factors. In the present study 22 patients declared the contact dermatitis, 16 patients declared the allergic rhinitis (AR) and 2 patients declared the diagnosed bronchial asthma (BA). Among them 7 (with AR) and 1 (with BA) described the severity as strong. The obtained results seem to support the observation of the authors.

In comparison performed by Simpson et al. AD patients colonized with *S. aureus* more often experience severe course of AD than non-colonized individuals and demonstrate the elevated levels of IgE and eosinophils in serum. Furthermore, the skin barrier of *S. aureus* colonized patients is significantly impaired with enhanced expression of adhesive molecules and increased permability what made skin much more prone to be colonized [29]. In our study patients were asked to evaluate the grade the intensity of AD flares at the moment of specimen collection. What is interesting, the severity of AD exacerbations described as “strong” was declared by 7 patients from *group I* vs 11 who declared the severity of AD flares ass “moderate” (39%). “Strong severity of AD was declared by 19% and 25% of patients from *group II* and *group III* respectively. None of the patients declared the AD severity as “mild”. The above results seem to be consistent with Alsterholm et al. [30] and Ogonowska et al. who observed, that especially persistent nasal *S. aureus* carriers expierience more severe AD according to SCORAD system [31]. Its worth mentioning, that the present study evaluation of nasal carriage was performed ones for each patient, therefore in *group III* of patients (nasal non-carriers) intermittent nasal carriage cannot be certainly excluded. To support the management of AD it would be appropriate to consider nasal colonization screening performed periodically in these group of patients.

Patients examined in the present study were also asked to rate the frequency of flares in previous 6 months. None of the patients declared less than 4 episodes. Patients from *group I* with strong form of AD declared the frequency as 5–6 episodes. Ogonowska et al., [31] observed, that the concomitant nasal and skin colonization in AD patients is comparatively high and nasal vestibule is considered as the potential reservoir of *S. aureus*, contributing to the recolonization of skin.

The nasal vestibule constitutes the reservoir of *S. aureus,* from which it spreads to other body sites and may cause multiple autoinfections [32, 33]**.** It is estimated, that 20–30% of healthy population representatives are persistent carriers of this bacterium [34]. The percentage of *S. aureus* carriers is higher among people with AD (38–82%) compared to healthy individuals (10–45%). In the current study nasal carriers constituted more than a half (53,1%) of the of examined patients (34/64) and them in 18 (18/34) the simultaneous presence of *S. aureus* on skin was confirmed. Breuer et al. isolated *S. aureus* from 94% of people with AD: from 11% exclusively from the skin, from 6% only from the nose, and from 77% from affected skin and nasal vestibule [35].

Nose (nasal vestibule) of most people with SSTI’s is commonly colonized with indistinguishable, closely or potentially related *S. aureus* [35]*.* Phage typing and serotyping results performed by Namura et al. and Hoeger et al. revealed significant similarities between *S. aureus* strains isolated from cutaneous lesions and nasal vestibule in 64% (7 out of 11) adult patients with AD and in 73% (30 out of 41) children with AD respectively [36, 37]. Hoeger et al. demonstrated also the presence of indistinguishable *S. aureus* isolated from affected skin and from nasal vestibule of children with AD and isolated from their mothers (in 38% of cases – 22 out of 58) [37]. Pascolini et al. evaluated the risk of transmission of the *S. aureus* colonizing nasal vestibule to the other skin sites of the same patient with using the PFGE technique to determine the relatedness of analyzed strains. All 47 strains isolated from cutaneous lesions were genetically related to the strains isolated simultaneously from nose [38]*.* Detailed analysis of *S. aureus* isolated from colonized individuals sharing the same habitat and remaining in close contact with patients with AD proved, that *S. aureus* easily transmits between those individuals contributing a carrier state [35, 39, 40]. Similar PFGE findings were observed by Bonness et al. who confirmed the relatedness of *S. aureus* isolated from nose and skin in 73% (11 out of 15) of patients with AD and suggested, that significant similarities between PFGE patterns of *S. aureus* isolated from AD patients and their family members may prove the recolonization with *S. aureus* as intra - familial transmission of *S. aureus*. The *S. aureus* skin recolonization may be associated with the pathogen maintaining the carrier state in the vestibule of the nose [41]. PFGE results obtained in our study demonstrated that skin and nasal vestibule were colonized with indistinguishable strains in 16 (16/18) of examined patients and in 2 (2/18) with non- related *S. aureus.* The results showed also non-clonal structure of *S. aureus* isolated from affected skin of patients with AD and also the lack of predominating genetic type of *S. aureus* infecting patients with AD in West Pomeranian region. It’s worth mentioning, that all patients examined in current study were during the targeted antimicrobial therapy without the implemented nasal decolonization procedures. Therefore, the recurrent exacerbations of AD symptoms were most probably associated with autoinfection with *S. aureus* in these patients.

In the current study isolated *S. aureus* showed the low rates of antimicrobial resistance to examined agents. Although an increasing rates of methicillin resistant *Staphylococcus aureus* (MRSA) colonizing outpatients are reported worldwide [42, 43] reaching approximately 7% within the total population, the skin of outpatients with AD is most frequently affected with methicillin susceptible *Staphylococcus aureus* (MSSA). Data concerning the rates of AD patients colonized with MRSA vary. Kędzierska et al. among 76 *S. aureus* isolated from patients with AD determined the resistance to methicillin only in one strain isolated from 9-year old boy with history of hospitalization and prolonged antibiotic therapy [44]. Low rates of MRSA was also confirmed by Niehbuhr et al. who determined the presence of methicillin resistance only in 3% of analyzed *S. aureus* isolated from AD patients [45]. Pascolini et al. among 113 *S. aureus* isolated from carriers and from their infected skin found the presence of methicillin resistance in 9 strains (7,9%) of which 6 were isolated from skin lesions [38]. In studies conducted by Hoeger et al. and L.S. Chiu et al. the lack of MRSA was confirmed among all tested strains isolated from AD [37, 46]. Nevertheless, Ching - Shen Tang et al. demonstrated as many as 30,8% of MRSA colonizing skin of 78 healthy children with AD and 60% of MRSA infecting skin of 20 children with AD. High percentage of MRSA colonizing different body sites in patients with AD is most probably closely associated with the selection of MRSA following prior therapy with beta-lactams [47]. It is worth remembering, that skin colonization with MRSA also follows previous hospitalization increasing the likelihood of contact with hospital strains [48] or may be associated with the direct contact and transmission of MRSA within community. In the present study all examined patients denied hospitalization within 6 months before specimen collection, were also neither related nor shared the same habitat, therefore the low rates of MRSA within the patients examined in the present study seem to support the above observation.

In the study the susceptibility to erythromycin and clindamycin of investigated *S. aureus* was also determined. MLSB – the acquired resistance to macrolides, lincosamides and streptogramin B results from the decreased binding of these antibiotics to the methylated 50S ribosomal subunit, an overlapping binding site for all three groups of antimicrobial agents [49]**.** Constitutive MLSB (MLSBc) phenotype is associated with the rRNA methylase constantly produced by the bacterial strain**,** whereas in the bacteria with inducible MLSB (MLSBi) the production of methylase is observed in the presence of an inducing agent [50]. The D-test method is reccomended to determine the inducible MLSB, due to fact that this type of resistance cannot be determined with standard susceptibility tests. D-test reveals the inducible resistance to clindamycin (MLSBi) when disc with clindamycin is placed in close proximity to erythromycin disc and D-shaped inhibited growth zone is flattened between erythromycin and clindamycin. This method allows to avoid the clinical failures of treatment with clindamycin the infections caused by MLSBi strains and to distinguish the MLSBi from MS phenotype –strains resistant to erythromycin and streptogramin B but susceptible to clindamycin [51]. The importance of the proper determination MLSB phenotype is worth to underline, since clindamycin is recommended to treat uncomplicated skin and soft tissue infections, particularly an alternative for the treatment of infections caused by MRSA [52]**.**

MLSB resistance phenotype was confirmed in 7 strains,among them 3 simultaneously showed methicillin resistance. Concomitant resistance to beta-lactams, macrolides and lincosamides significantly compromises therapy leaving not many treatment options for therapy in community [53–55].

Although topical skin decolonization with antimicrobial agents may reduce the risk of subsequent SSTI’s [34, 35] and may be an important element of medical strategy in patients with AD. However Polish Society of Atopic Diseases does not recommend the prolonged topical use of antibiotics and oral antimicrobial therapy, short courses of oral antibiotics, such as cephalosporins, may be implemented in patients with clinical signs of bacterial infection. Other studies have shown that treatment with oral flucloxacillin or cefuroxime caused a significant reduction in *S. aureus* skin colonization in children. However no significant relief of AD symptoms was observed. Soon after the treatment, recolonization with *S. aureus* was observed [56] and exacerbation of AD symptoms were assumed [57]. Prolonged antimicrobial treatment entails also the risk of adverse effects such as skin microbiota disruption, contact dermatitis or selection of resistant bacteria, including MRSA.

Due to the fact that oral antimicrobial agents poorly penetrate the nasal mucosa, an oral treatment with topical application of mupirocin may be an improvement of carrier state eradication outcomes [12]. In the present study vast majority of examined strains isolated from nasal vestibule (79/86) showed susceptibility to mupirocin and the obtained result is close to ones obtained for *S. aureus* isolated from patients in Europe (6,6%) [58]. Mupirocin has been approved for eradication of MRSA and MSSA nasal carriage, however, the increasing prevalence of resistance to this antimicrobial agent among *S. aureus* is observed worldwide. In our study the lack of methicillin resistance in mupirocin resistant isolates was confirmed. Therefore overuse or uncontrolled use of mupirocin may lead to increased emergence of resistant strains and hence to reduction of the likelihood of *S. aureus* successful eradication.

It’s important to underline that antimicrobial therapy should be considered only in exacerbation of AD symptoms and complications following multiple and recurrent bacterial skin infections. The immunization with autovaccines composed of unique *S. aureus* antigens that aim the skin beneficial bacteria remain unharmed, is considered as an alternative in AD management [59–61].

Consequently, management of AD complicated by *S. aureus* colonization should also consider the eradication of *S. aureus* from nose. With the restoration of skin structure and its function it may decrease the risk of autoinfection and the frequency of AD exacerbations significantly improving the severity of AD.

## Data Availability

All data generated or analysed during this study are included in this published article. Data sharing is not applicable to this article as no datasets were generated or analysed during the current study. *S. aureus* isolates used in the current study are the part of the collection belonging to the Independent Laboratory of Medical Microbiology, Pomeranian Medical University, Szczecin, Poland.
